# Double Ureter: Incidence, Types, and Its Applied Significance—A Cadaveric Study

**DOI:** 10.7759/cureus.7760

**Published:** 2020-04-21

**Authors:** Sangeetha Arumugam, Nandha Kumar Subbiah, Arathi Mariappan Senthiappan

**Affiliations:** 1 Anatomy, Katuri Medical College and Hospital, Guntur, IND; 2 Anatomy, All India Institute of Medical Sciences, Mangalagiri, IND; 3 Anatomy, Chettinad Hospital and Research Institute, Chennai, IND

**Keywords:** double ureter, complete double ureter, ureteroureteric reflux, incomplete double ureter

## Abstract

Introduction

Congenital ureter anomalies such as double ureters are uncommon developmental anomalies of the renal system. An abnormal branching pattern of ureteric bud results in the formation of double ureter. This study examined the incidence of double ureter in cadavers of a South Indian population.

Methods

A total of 50 kidney and ureter specimens were carefully dissected out of the posterior abdominal wall and examined for the presence and subtype of double ureter.

Results

Of 50 kidneys, three (6%) specimens showed an incomplete double ureter, two on the right kidney and one on the left. In all three specimens, the double ureter fused at different levels to form a single ureter opening into the bladder.

Conclusions

The prevalence of incomplete double ureter is higher in this study compared with that in previous cadaveric studies. Ureteral injuries are a frequent complication of abdominal and pelvic surgeries. Hence, awareness about the types and varieties of double ureter will aid radiologists and surgeons in interpreting and diagnosing urological images and preventing accidental injury while performing surgery.

## Introduction

The ureters are muscular ducts, around 25-30 cm in length, with narrow lumina. These ureters drain urine from the corresponding kidney to the urinary bladder. The ureters have two segments: abdominal and pelvic. The abdominal segments of the ureters adhere closely to the parietal peritoneum and are retroperitoneal throughout their course, and the pelvic segments enter the pelvis by passing over the pelvic brim at the bifurcation of the common iliac arteries before entering the urinary bladder [1]. Congenital anomalies of the kidney and urinary tract, including double ureter, constitute 20% to 30% of all prenatal anomalies. Double ureter may present as either complete or incomplete/partial duplication [2,3]. Clinically, patients with a double ureter may be asymptomatic or may present with hematuria or abdominal or ﬂank pain and be predisposed to ureteral obstruction, ureteroureteric reﬂux, and recurrent urinary infections [4,5].

Ureteral injury is a common complication of open or laparoscopic surgical procedures involving the abdomen and pelvic region. The occurrence of such ureteral injuries can be prevented by prior imaging of the abdomen and pelvis, as well as examining the ureter. In-depth knowledge of the normal and abnormal patterns of the ureter is a prerequisite for both radiologists and surgeons to plan any surgical procedure. Many radiologists have reported double ureters after performing excretory urethrograms [6]. However, cadaveric studies on ureteric variations are seldom undertaken. Hence, this cadaveric study was performed to determine the prevalence of double ureters in a South Indian population and to discuss their clinical importance.

## Materials and methods

This study was approved by the Ethics Committee of Katuri Medical College, Guntur, India. Kidneys with intact ureters and bladder were dissected and removed from cadavers of 25 South Indian individuals of either sex, following the guidelines outlined by Cunningham’s Manual of Practical Anatomy [7]. The kidneys, along with the ureters and bladder, were washed thoroughly in running water after removal and were stored in 10% formalin. Morphologically damaged kidneys were excluded from the study. Kidneys with double ureters and their subtypes were described and photographed. The results were tabulated and summarized in Microsoft Excel and are shown in Table 1.

**Table 1:** Percentage of complete and incomplete ureters

Ureter (50)	Complete	Incomplete	Percentage
Right ureter (25)	0	02	4%
Left ureter (25)	0	01	2%

## Results

**Figure 1:**
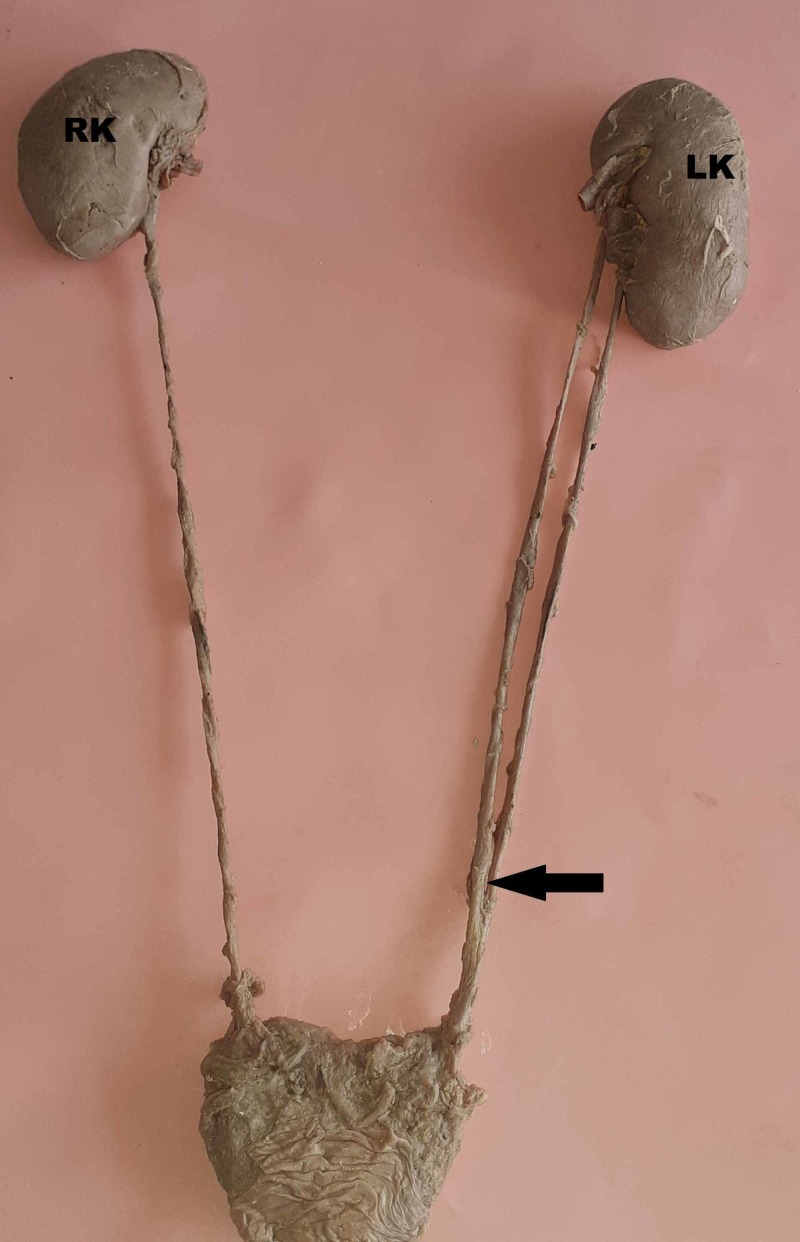
Left kidney with an incomplete double ureter RK, right kidney; LK, left kidney

**Figure 2:**
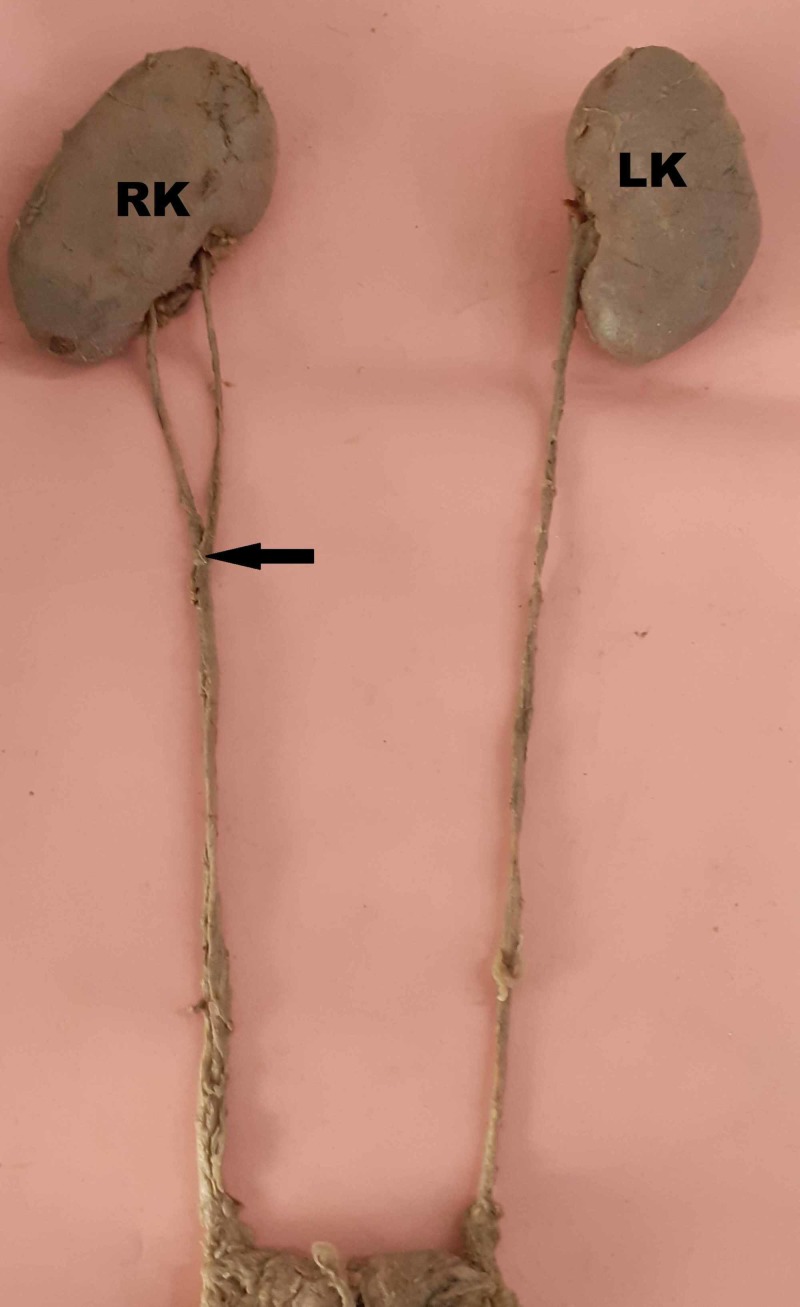
Right kidney with an incomplete double ureter RK, right kidney; LK, left kidney

**Figure 3:**
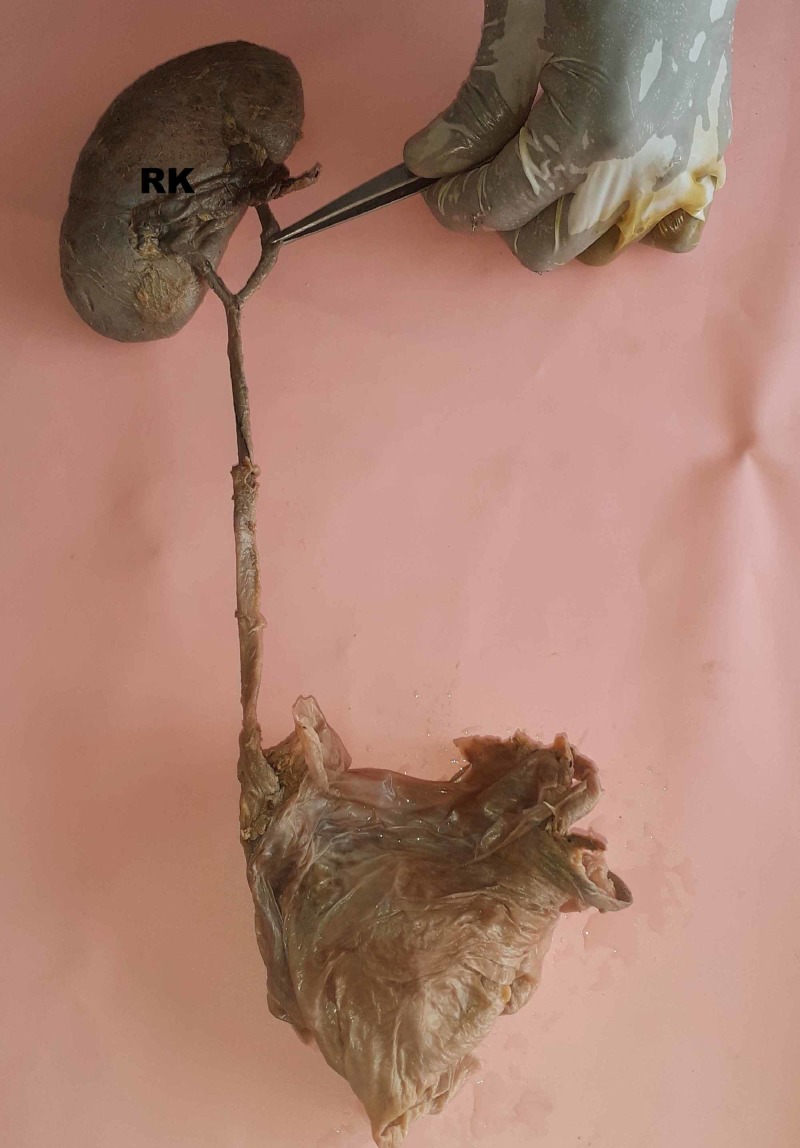
Right kidney with incomplete double ureter fusing at the lower pole of the kidney RK, right kidney

Upon examination of 50 kidneys, 47 (94%) had a normal single ureter arising from the renal pelvis and opening into the urinary bladder. The remaining three (6%) specimens showed variations in ureters (Table 1). In one pair of kidneys, the left kidney had double ureters arising from the upper and lower poles of the renal pelvis that joined with each other to form a single ureter (Y-shape) distally before opening into the urinary bladder (Figure 1). In the second pair, double ureters emerging from the right kidney were seen fusing midway between the kidney and bladder (Figure 2). In the third specimen, double ureters arising from the right kidney formed a single ureter a few centimeters away from the renal pelvis (Figure 3). All double ureters were unilateral, were incomplete in presentation, and opened through a single ureteric orifice into the urinary bladder.

## Discussion

The urinary system develops from two sources: metanephric blastema and mesonephric duct. Ureteric bud arises as a diverticulum from the caudal end of the mesonephric duct. This diverticulum elongates and later fuses with the metanephric blastema to form renal pelvis, which further divides into major and minor calyces [2,4]. Double ureter is caused by abnormalities in the branching pattern of the ureteric bud. In the case of complete duplication, the ureteral bud arises twice, resulting in a double ureter with a double opening into the urinary bladder. In rare cases, one of the ureters can open into sites other than the urinary bladder, such as the vagina, seminal vesicle, urethra, prostate, epididymis, or vas deferens. This condition is called ectopic ureter. Incomplete duplication is due to splitting of the ureteric bud anywhere along its course to its termination into the metanephric blastema. The duplicated ureters unite at a variable distance away from the kidney, and only one ureteric orifice is seen in the bladder on that side. If the ureteric bud bifurcates after fusing with metanephric tissue, it results in a double pelvis and double ureter [8].

Autopsy studies have suggested that the incidence of unilateral bifid ureter is 1 in 125 cases (0.8%) [1]. Deka and Saikia reported double ureters in 1.67% of 60 specimens, and, incidentally, all were on the left side [9]. Choudhary et al. studied 32 specimens, of which two (6.25%) kidneys showed unilateral incomplete duplication [10]. Roy et al. reported double ureters in 0.64% of 156 kidney specimens [11]. According to Siomou et al., double ureters have a common unilateral presentation [8]. In this study, we observed an incomplete double ureter in three kidneys (6%) of 50 specimens, two on the right kidney and one on the left. This is the second-highest prevalence of double ureter among the studies reported above; thus, we infer that the prevalence of double ureter is higher in South Indian populations. Moreover, all double ureters were unilateral, affecting either the right or left kidney. The prevalence of double ureters reported by various studies, including this study, is shown in Table 2. Dähnert studied excretory urograms and reported that incomplete duplication of the ureter was three-fold more common than complete duplication [12].

**Table 2:** Incomplete double ureter reported by various authors and this study

Authors	Incomplete double ureter/total specimens	Percentage of incidence
Deka and Saikia [9]	1/60	1.67%
Choudhary et al [10]	2/32	6.25%
Roy et al [11]	1/156	0.64%
Prakash et al [13]	2/50	4%
This study	3/50	6%

Prakash et al. studied radiological and cadaveric specimens in 50 pairs of kidneys and also reported that incomplete duplication was more common than complete duplication [13]. A similar finding was observed in this study; all three kidneys observed showed incomplete double ureters. Based on the level of union of double ureters, Dorko et al. classified double ureters as proximal (ureter fissus proximalis) and distal (ureter fissus distalis) [14]. In this study, two ureters united proximally (Figures 2, 3) and one distally on the left side (Figure 1).

There are three constrictions along the length of the normal ureter that predispose the ureter to stagnation of urine flow and occlude the passage of renal calculi [1]. In the case of an incomplete double ureter, the angled point of union of the two ureters creates a fourth constriction that can further obstruct normal flow, leading to reverse urine flow and associated complications such as hydronephrosis. Reflux is one of the most common complications associated with double ureter [15]. Studies have shown that patients with an incomplete double ureter are predisposed to ureteroureteric reflux, whereas a complete double ureter is usually associated with vesicoureteric reflux. Secondary to reflux, urinary tract infection and obstructive uropathy may occur [16,17]. Hence, we conclude that incomplete double ureters are diagnostically more significant than complete double ureters due to the complications associated with them.

Ureteric injuries are a potential complication of any open or laparoscopic surgical procedure involving the abdomen and pelvis [18]. Varlatzidou et al reported the presence of a complete double ureter as an incidental finding during surgery for colorectal cancer in a female patient [5]. Kelly et al. reported double ureters as an incidental finding in women presenting with vesicovaginal fistula for surgical repair [19]. A study by Kulkarni et al. revealed that duplication of ureters is more common in females [20]. In this study, we observed double ureter in one male and two female cadavers. Thus, double ureters are more common in females and are often an incidental finding during surgery. The presence of an incomplete double ureter increases the likelihood of ureteral injury during surgery and associated secondary complications. These undesired complications can be avoided by the use of preoperative radiographic imaging, such as intravenous urography or CT scans, for any open or laparoscopic surgical procedure involving the kidneys, including colorectal, general, vascular, and gynecological surgeries. Radiologists must be familiar with the different types and levels of fusion of incomplete double ureters before interpreting the images.

## Conclusions

Double ureter is a developmental anomaly affecting the urinary system. This cadaveric study was performed in 50 kidney and ureter specimens to determine the incidence of double ureters in a South Indian population. The incomplete double ureter was observed unilaterally in three kidneys (6%) of 50 specimens, two on the right kidney and one on the left. The angled point of union of incomplete double ureter predisposes to complications such as ureteroureteric reflux. The presence of an incomplete double ureter increases the possibility of ureteral injury during surgery and misinterpretation of radiological images. Hence, radiologists and surgeons must be familiar with the complete/incomplete double ureter and its subtypes.
